# Influence Mechanism of Waterborne Polyurethane on the Properties of Emulsified Asphalt

**DOI:** 10.3390/ma18143280

**Published:** 2025-07-11

**Authors:** Jian Tan, Shuguang Hou, Rui Jin, Xiao Zhong, Xiaoxi Zou

**Affiliations:** 1 Zhaotong Yizhao Expressway Investment and Development Co., Ltd., Zhaotong 657000, China; 2 College of Transportation Engineering, Nanjing Tech University, Nanjing 211816, China; jrzs@njtech.edu.cn; 3 Zhaotong Transportation Bureau, Zhaotong 657099, China

**Keywords:** modified emulsified asphalt, waterborne polyurethane, ionic emulsifiers, influence mechanism, chemical composition, microphase structure

## Abstract

To elucidate the modification mechanism of waterborne polyurethane (WPU) on emulsified asphalt, anionic and cationic WPUs are utilized as modifiers. As well, their effects on physical properties, microstructure, and compatibility are characterized using basic performance tests, Fourier transform infrared spectroscopy (FTIR), and atomic force microscopy (AFM). The results show that WPU-modified emulsified asphalt exhibited a higher softening point, reduced penetration, and decreased ductility, suggesting enhanced high-temperature stability but diminished low-temperature flexibility. Among all samples, the combination of cationic WPU with cationic emulsified asphalt shows the highest softening point (54.1 °C), whereas cationic emulsified asphalt alone exhibits the lowest one (52.9 °C). Anionic emulsified asphalt demonstrates the highest penetration (79 mm), while non-ionic WPU combined with cationic emulsified asphalt shows the lowest one (59.3 mm). The ductility decreases from 90.3 cm to 28.7 cm. The storage stability varies with WPU ion type. Cationic WPU-modified samples showed the poorest storage stability (0.7% residue), while anionic-modified samples exhibit the best one (0.4% residue). FTIR analysis confirms the presence of characteristic WPU absorption peaks, indicating that physical blending occurs, and chemical interaction is limited. AFM observations reveal that anionic WPUs provide superior compatibility, forming fine, uniformly distributed particles with the lowest surface roughness (5.655 nm). In contrast, cationic WPUs form chain-like structures that cure effectively but exhibit poor dispersion. This study provides a basis for the development of high-performance WPU-modified emulsified asphalt.

## 1. Introduction

The cold recycling of asphalt pavement using emulsified asphalt offers several advantages, including high reclaimed asphalt pavement (RAP) utilization, construction at ambient temperature, extended construction seasons, and minimal environmental pollution. Recognized as a green technology with low emissions and minimal environmental impact, cold recycling aligns with China’s strategic goals of carbon peaking and carbon neutrality, and is receiving increasing attention in the highway engineering field. Its application in engineering practice is expanding. As the primary binder in cold recycling, emulsified asphalt is widely used in asphalt pavement maintenance and repair [1]. Through the action of surfactants, emulsified asphalt forms a stable emulsion system in water, improving both its workability and environmental properties.

However, both practical experience and research demonstrated that traditional emulsified asphalt cold-recycled pavements were associated with several critical limitations, including inadequate mechanical performance, low toughness, accelerated deterioration, and shorter service life [2]. These pavements are prone to cracking and loosening, which undermine overall mechanical properties and road performance [3], ultimately reducing their service life. Therefore, the development of high-performance modified emulsified asphalt has become a critical focus in pavement engineering.

Extensive studies were conducted to enhance performance, aging resistance, and storage stability of emulsified asphalt. Among various modifiers, waterborne polyurethane (WPU) has gained some attention due to its favorable mechanical strength, weather resistance, and environmental friendliness. WPU has been shown to improve the rheological properties of asphalt, particularly its high-temperature deformation resistance [4,5,6]. Zhang et al. [7] reported that 15% WPU content offers optimal performance and significantly improves storage stability. Hesami et al. [8] concluded that WPU enhanced the ductility but reduced the adhesion of emulsified asphalt, and the modification process was predominantly physical. The WPU content of less than 6% was therefore recommended. Similarly, Zhao et al. [9] observed a positive correlation between WPU dosage and performance when the dosage remains under 9 wt%. In contrast, Mu et al. [10] argued that limited compatibility between WPU and asphalt led to inconsistent modification effects. Therefore, further research was required to better understand the compatibility, structural integrity, and microstructural evolution of WPU-modified emulsified asphalt [11].

WPU has been classified into cationic, anionic, and non-ionic types [12]. The stability of emulsified asphalt is influenced differently by emulsifiers of various ionic types [13]. Anionic polyurethane has been reported to enhance the rheological behavior of emulsified asphalt, shifting its characteristics from viscous to elastic performance [14]. FTIR analysis revealed that the modification of cationic emulsified asphalt by WPU primarily involved physical blending [15]. Mu et al. [16] examined the performance of cationic WPU-modified emulsified asphalt across different temperatures and identified 110 °C as the optimal evaporation residue temperature, where FTIR absorption peak was the most intense and key performance indicators—penetration, softening point, and ductility—were significantly improved. Although various modification effects were observed among different WPU ionic types [17], the underlying mechanisms remain insufficiently studied [18,19], particularly concerning the compatibility behavior between emulsified asphalt and WPU with different ionic natures.

The incorporation of WPU into emulsified asphalt effectively improves its performance. However, the poor compatibility between WPU and asphalt limited further enhancement of modification effects. To address this issue, researchers commonly regulated WPU structure by introducing ionic groups. Most existing studies focused on modification mechanisms of cationic WPU, while limited attention was given to anionic and non-ionic types. The specific roles and comparative effects of different WPU types on emulsified asphalt remained unclear, and a systematic understanding of their interaction mechanisms, interfacial behavior, and impact on asphalt microstructure is still lacking. Therefore, this study aimed to develop WPU-modified emulsified asphalt using different ionic types of WPU and to clarify their modification effects, revealing the mechanisms by which WPU influences the performance and structure of emulsified asphalt.

In this study, anionic, cationic, and non-ionic WPUs were incorporated into anionic and cationic emulsified asphalt systems to prepare modified samples. These samples were first evaluated for basic metrics, including penetration, softening point, ductility and storage stability. FTIR analysis was then conducted to investigate the effects of WPU type on chemical composition and key functional groups, providing insights into the physical and chemical interactions between WPU and asphalt. Finally, AFM tests were used to examine the microstructural characteristics of modified samples, revealing the compatibility mechanism and microstructural evolution of WPU-modified emulsified asphalt. A comprehensive framework linking performance, structure, and mechanism was established, clarifying the regulatory role of WPU ionic structure in interfacial compatibility and modification effectiveness. This work provides theoretical support and technical guidance for the design and optimization of high-performance WPU-modified emulsified asphalt materials.

## 2. Materials and Methods

### 2.1. Materials

#### 2.1.1. Base Asphalt

Emulsified asphalt was prepared using 70# base asphalt with a penetration grade of 60–80, and its basic properties were tested according to JTG E20-2011 [20]. The results as shown in Table 1 indicated that all material properties met the requirements of JTG F40-2004 [21]. The test methods used in Table 1 were all conducted in accordance with JTG E20-2011 [20]. The asphalt used in this study was supplied by Zhonghai Asphalt Co., Ltd., Binzhou, Shandong, China.

#### 2.1.2. Anionic/Cationic Emulsifiers

Slow-cracking fast-setting cationic emulsifier and slow-cracking fast-setting anionic emulsifier produced by Rongcheng Road and Bridge Co., Ltd., Nantong, China, were selected, and the performance indicators are listed in Table 2.

#### 2.1.3. Experimental Water

The water used in this experiment was laboratory tap water without any impurity. The main water quality parameters were as follows. The pH value ranged from 7.5 to 8.5, electrical conductivity ranged from 5 to 8 μS/cm, hardness ranged from 0.1 to 0.3 mmol/L, and chloride ion concentration ranged from 10 to 30 mg/L. This water quality ensured that there was no additional interference in the emulsification process.

#### 2.1.4. Hydrochloric Acid Modifier

Cationic emulsifiers require an acidic environment when used as modifiers, while anionic emulsifiers require an alkaline environment. Therefore, when using cationic emulsifiers, hydrochloric acid was used to adjust the pH value to 2~3, and when using anionic emulsifiers, alkali was used to adjust the pH value to 12~13. The basic indicators of hydrochloric acid and sodium hydroxide are shown in Table 3. Both materials were purchased from Sinopharm Chemical Reagent Co., Ltd., Nanjing, China.

#### 2.1.5. Emulsifier 

Two types of anionic and cationic emulsified asphalts were used, and their basic performance indicators are shown in Table 4. They were supplied by Yutuo Chemical Co., Ltd., Panjin, Liaoning, China.

#### 2.1.6. Different Ionic WPUs

The performance parameters of different ionic WPUs are presented in Table 5. The waterborne polyurethane was purchased from Shanghai Sisheng Polymer Materials Co., Ltd., Shanghai, China. The content of each type of polyurethane in this study was set at 4 wt%.

### 2.2. Sample Preparation

#### 2.2.1. Preparation of Matrix Emulsified Asphalt

The emulsification equipment selected for this experiment was the JM-1 small emulsifier from Wuxi Petroleum Instrument Equipment Co., Ltd., Wuxi, China. Sample preparation was performed as follows. To ensure the reliability and reproducibility of test results, parallel experiments were carried out, and no fewer than three effective samples were tested for each group. The allowable error was limited to 15%. If this threshold was exceeded, the samples were re-prepared and retested. The procedure was as follows:

(1) Heating of asphalt

To ensure proper flowability, base asphalt, which was solid at room temperature, was preheated in an oven at 140 °C. This step allowed asphalt to flow smoothly through the colloid mill during its emulsification.

(2) Preparation of soap solution

The soap solution was prepared by dissolving the anionic/cationic emulsifier in water in a predetermined ratio. The solution temperature of soap solution was controlled at approximately 60 °C.

(3) Preparation of emulsified asphalt

Prior to emulsification, hot water (60 °C) was circulated through colloid mill to minimize heat loss during its emulsification. Once preheating was complete, soap solution was cycled into colloid mill. After no significant bubbles were generated, the heated asphalt was slowly added. The mixture was sheared for 1 min, and then the three-way valve was opened to release emulsified asphalt.

(4) Storage of emulsified asphalt

Immediately after preparation, emulsified asphalt was allowed to cool to room temperature while being gently stirred with a glass rod. After cooling, it was filtered using a 1.18 mm filter screen. Once filtration was completed, it was sealed for storage.

#### 2.2.2. Preparation of WPU Modified Emulsified Asphalt

The preparation equipment for WPU-modified emulsified asphalt was selected to be an FM 300 intermittent high-speed shear disperser from Shanghai Fluke Company, Shanghai, China.

The preparation methods for modified emulsified asphalt are generally categorized into two approaches, including modification prior to emulsification and emulsification followed by modification. In this study, the former approach was initially used. The soap solution was prepared and emulsified by a colloid mill, after which WPU was added, after which WPU was added, followed by the gradual incorporation of heated base asphalt. However, emulsified asphalt showed poor flowability and solidified after being left for a period, which led to a poor emulsification effect. Therefore, the method of emulsification followed by modification was adopted. Base emulsified asphalt was first prepared. After that, WPU and emulsified asphalt were blended using a high-speed shear mixer. During this process, a glass rod was used for slowly stirring until the sample cooled to room temperature, after which it was sealed and stored.

### 2.3. Test Methods

#### 2.3.1. Basic Performance Test

Basic properties of prepared WPU-modified emulsified asphalt were evaluated according to JTG E20-2011 [20], including penetration, ductility, and softening point.

#### 2.3.2. FTIR Test

To analyze the effect of WPU on primary chemical components and functional groups of emulsified asphalt, samples were prepared by depositing a thin film onto specialized silicon wafers (10 mm × 10 mm) using a pipette, and the film thickness was controlled to less than 10 mm. After film formation, infrared spectroscopy tests were conducted. FTIR spectroscopy test was performed using a Thermo Scientific Nicolet iS50 FTIR spectrometer (Thermo Fisher Scientific, Madison, WI, USA). The front surface was selected for testing, and the test mode was set to ATR with absorbance. Each sample and background were scanned 32 times. The resolution was set at 4 cm^−1^, with a gain of 1.000, mirror speed of 0.4747, and an aperture of 100. The spectral range was set from 4000 cm^−1^ to 400 cm^−1^.

#### 2.3.3. AFM Test

To investigate the effect of WPU on micromorphology and microstructure of emulsified asphalt, samples were prepared by pipetting the material onto specialized silicon wafers (10 mm × 10 mm) using a pipette, and each sample thickness was controlled to less than 10 mm. After curing, AFM analysis was performed using a Dimension FastScan Atomic Force Microscope (Bruker Corporation, Billerica, MA, USA). The micro-cantilever was characterized by a tip radius of 650 nm, a length of 115 μm, and a width of 25 μm. The scanning area was set to 10 μm × 10 μm, with a resolution of 256 × 256. The tip operated at a frequency of 70 kHz and possessed an elasticity coefficient of 0.4 N/m.

## 3. Results and Discussion

### 3.1. WPU-Modified Emulsified Asphalt Basic Properties

To evaluate the effect of different types of WPU on basic properties of emulsified asphalt, a series of fundamental property tests were performed on the prepared samples. The corresponding results are presented in Table 6. For clarity, the abbreviations used are based on the first letters of sample names.

In Table 6, the residual amount on the sieve for all systems remains very low (0.002–0.015%), indicating that emulsified asphalt particles are fine and the sieve loss is minimal, reflecting better emulsion quality and system stability. The viscosity ranges from 12 Pa.s to 17 Pa.s. After modification by WPU, the standard viscosity of emulsified asphalt increases to varying extents. This suggests that the incorporation of WPU enhances the viscosity of emulsified asphalt, thereby improving its adhesion and high-temperature stability.

As shown in Table 6, the penetration values of four WPU-modified emulsified asphalt samples decrease when compared to that of base emulsified asphalt, indicating an improvement in high-temperature stability. This trend corresponds to the observed increase in standard viscosity. The decreased penetration correlates with increased viscosity, consequently elevating the softening point while reducing ductility at 15 °C. Compared to the base cationic or anionic emulsified asphalt, WPU-modified samples demonstrate lower storage stability, primarily affected by the pH and particle size of WPU. WPU is slightly alkaline, and when mixed with acidic emulsified asphalt, heat is generated, which accelerates moisture evaporation and decreases stability [22]. Larger particle sizes promote faster settling, further compromising stability. The use of anionic emulsifiers helps reduce particle size and improve stability. However, the dosage must be optimized, as both insufficient and excessive amounts of WPU negatively affect performance of modified emulsified asphalt.

Compared to the control sample (unmodified cationic emulsified asphalt), the cationic WPU-modified emulsified asphalt is characterized by a 19.6% reduction in penetration, a 2.27% increase in softening point, and a 17.7% reduction in ductility. In the non-ionic WPU-modified system, the penetration is reduced by 20.9%, the softening point is reduced by 1.7%, and the ductility decreases by 30.7%. Overall, WPU modification improves the hardness and high-temperature stability of emulsified asphalt but compromises its low-temperature toughness. Among the two, cationic WPU modification provides an improved rutting resistance while maintaining a certain level of low-temperature flexibility, making it suitable for regions with wide temperature fluctuations. In contrast, non-ionic WPU modification produces a harder emulsified asphalt with improved high-temperature stability but increases the susceptibility to low-temperature cracking. This is more appropriate for heavy-load environment at high temperature, but may increase cracking risk under cold conditions.

Compared to anionic emulsified asphalt, the penetration of anionic WPU-modified emulsified asphalt decreases by 11.4%, the softening point increases by 1.7%, and the ductility decreases by 50.5%. For non-ionic WPU-modified anionic emulsified asphalt, the penetration is reduced by 19.9%, the softening point is increased by 1.5%, and the ductility is decreased by 68.2%. Overall, WPU modification enhances the hardness and high-temperature stability of emulsified asphalt, while significantly compromising its low-temperature flexibility—particularly in the non-ionic system, where the reduction in ductility is more pronounced than in the anionic system. The anionic WPU-modified asphalt demonstrates an increased hardness and softening point, yet the ductility decreases by 50.5%, indicating the reduced low-temperature crack resistance. The non-ionic WPU-modified asphalt shows even greater hardness (19.9% reduction in penetration) and a 68.2% reduction in ductility, resulting in increased brittleness at low temperatures and making it more suitable for high-temperature applications. Although the anionic WPU-modified system demonstrates slightly better low-temperature crack resistance, further improvements remain necessary.

In summary, WPU modification enhances the hardness and high-temperature stability of emulsified asphalt but reduces its low-temperature ductility and storage stability. After modification, penetration values are reduced, the softening point increases, and the ductility decreases, indicating rutting resistance is improved but low-temperature ductility is diminished. Among the modified systems, cationic WPU-modified emulsified asphalt achieves a balance between high-temperature stability and moderate low-temperature flexibility, rendering it suitable for regions with large temperature fluctuations. The non-ionic WPU-modified system exhibits an increased hardness but suffers from pronounced low-temperature brittleness, rendering it more suitable for heavy-load applications in high-temperature environments. The storage stability generally decreases in all WPU-modified emulsified asphalt samples. Additionally, storage stability is generally reduced across all WPU-modified emulsified asphalts. Specifically, the cationic WPU-modified emulsified asphalt shows the lowest storage stability (0.7% sieve residue), while the anionic WPU-modified sample exhibits the highest stability (0.4% sieve residue). Overall, the anionic WPU system performs slightly better under low-temperature conditions but still requires further optimization to enhance crack resistance and storage stability.

### 3.2. Effect of WPU on Chemical Composition and Functional Groups of Emulsified Asphalt

WPU resin consists of two main components of polyisocyanates and polyether/polyester polyols [23]. It readily reacts with phenols, anhydrides, carboxylic acids, and even water present in emulsified asphalt, leading to the formation of polyurethane carbamate acid (Equation (1)). Isocyanates also react with phenols to generate carboxylic acids, which subsequently undergo further reactions to form anhydrides (Equation (2)). These anhydrides react with water to produce amides and carbon dioxide. When aromatic carboxylic acids are present in asphalt or aromatic groups exist within the isocyanates, such stable products as anhydrides and ureas are formed (Equation (3)). In addition, isocyanates react with anhydrides to yield acylamides (Equation (4)).



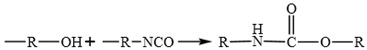

(1)




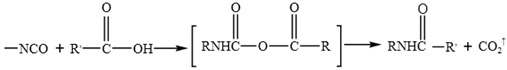

(2)




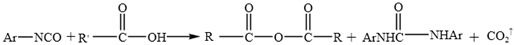

(3)




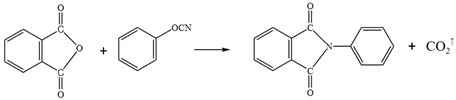

(4)


**Figure 1:**
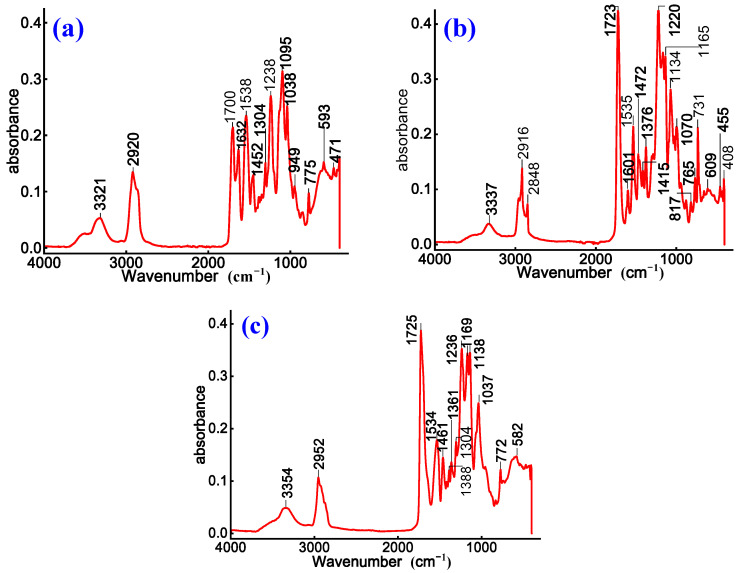
Infrared spectra of (**a**) non-ionic WPU, (**b**) cationic WPU, and (**c**) anionic WPU.

To investigate the curing behavior of WPU in emulsified asphalt and its interaction with asphalt matrix, FTIR is employed to analyze cationic and anionic emulsified asphalt, non-ionic WPU-modified emulsified asphalt, as well as cationic and anionic WPU-modified cationic and anionic emulsified asphalt. The functional groups and their variations in these blended systems are identified by examining characteristic peak positions and intensity changes intensity in the infrared spectra of each sample. Furthermore, FTIR is also used to characterize chemical structure and ionic properties of cationic, anionic, and non-ionic WPUs as shown in Figure 1.

As shown in Figure 1, the infrared spectra of cationic, anionic, and non-ionic WPUs are characterized by absorption peaks corresponding to carbamate (-NHCOO-) and ester (C=O) groups. The N-H and O-H stretching vibrations observed at 3321–3337 cm^−1^ appear more intense in non-ionic WPU, whereas the peak of anionic WPU broadens due to the presence of carboxyl and sulfonic acid groups. These variations in hydrogen bond interactions may influence the stability of WPU within the emulsified asphalt system. The enhanced hydrogen bonding in non-ionic WPU is considered to improve emulsion cohesion, whereas polar functional groups in anionic WPU are thought to affect the dispersion through electrostatic interactions.

The C-H stretching vibrations at 2920–2952 cm^−1^ correspond to the predominant soft segment structure in WPU. From Figure 1a, non-ionic WPU exhibits a stronger peak because of the presence of polyether or polyester segments, while cationic and anionic WPUs display a marginal reduction in peak intensity, which is attributed to the influence of polar groups. These differences in soft segment composition are considered to affect the compatibility and stability between WPUs and asphalt. Non-ionic WPU is considered to enhance wettability and dispersibility, while cationic and anionic WPUs may modify emulsification performance via polar group interactions.

In Figure 1, the C=O stretching vibration at 1700–1725 cm^−1^ is attributed to an ester functional group, with a slight red shift observed in cationic WPU. This shift is considered indicative of stronger interactions with acidic emulsifiers in emulsified asphalt, affecting the stability of emulsion, while anionic WPU interacts with alkaline components in asphalt, influencing the emulsification effect [24]. The benzene ring skeletal vibrations at 1534–1601 cm^−1^ are detected in all three samples but may be more pronounced in anionic WPU. This enhancement is thought to increase the rigidity of emulsified asphalt film, improving mechanical strength but potentially decreasing flexibility.

**Figure 2:**
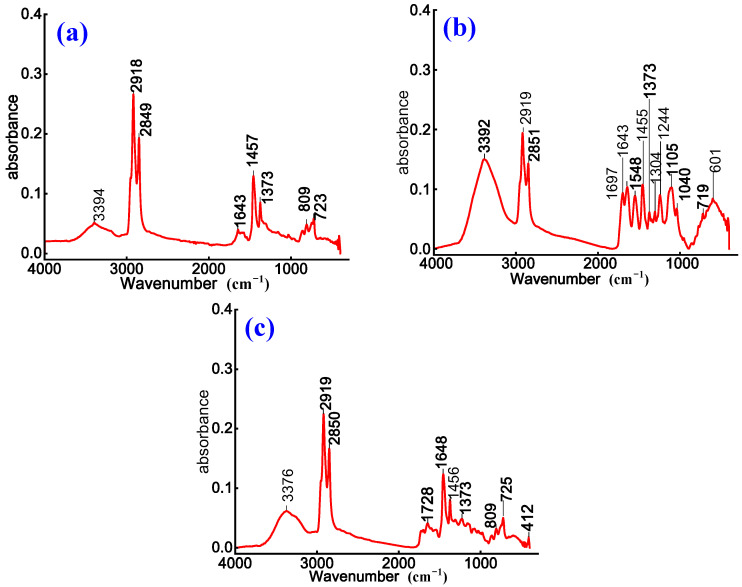
Infrared spectra of (**a**) cationic, (**b**) non-ionic WPU-modified cationic, and (**c**) cationic WPU-modified cationic emulsified asphalt.

To investigate chemical structural evolution and modification effects of cationic emulsified asphalt, non-ionic WPU-modified cationic emulsified asphalt, and cationic WPU-modified cationic emulsified asphalt, FTIR characterization is conducted on prepared samples. The corresponding results are presented in Figure 2.

As shown in Figure 2, the absorption peaks at 3394 cm^−1^, 3392 cm^−1^, and 3376 cm^−1^ are attributed to N–H stretching vibrations of quaternary ammonium salts and O–H stretching vibrations of emulsified asphalt. This enhancement suggests that the introduction of WPU increases hydrogen bond interactions, thereby strengthening the association between emulsified particles and ions, improving the stability of emulsified system [25]. The ester C=O absorption peaks at 1697 cm^−1^ in Figure 2b and 1728 cm^−1^ in Figure 2c, along with the amide C=O peak at 1648 cm^−1^ in Figure 2c indicate that WPU chemically reacts with asphalt, leading to the formation of new ester and amide bonds [26]. These reactions improve the stability of polyurethane within asphalt matrix, with a more pronounced effect observed in the cationic WPU-modified system.

In Figure 2b, the C–O stretching vibration at 1244 cm^−1^, along with ether C–O–C and alcohol C–O stretching vibrations at 1105 cm^−1^ and 1040 cm^−1^, respectively, suggest that the polyether or polyester segments in WPU’s soft phase affect the flexibility of emulsified asphalt. Among these, non-ionic WPU exhibits a more significant contribution, enhancing both the ductility and aging resistance of asphalt. The aromatic ring C-H out-of-plane bending vibrations at 900–700 cm^−1^ and long-chain methylene in-plane rocking vibration at 723 cm^−1^ in Figure 2a further confirm the dispersion of WPU within the asphalt matrix. Additionally, the C-N and O-H out-of-plane bending vibration at 601 cm^−1^ in Figure 2b indicates the presence of amide bonds and carboxyl groups, which influence the interfacial interactions of emulsified asphalt.

Overall, the formations of amide and carboxylic ester groups are more pronounced in cationic WPU-modified asphalt, indicating stronger chemical interactions that improve emulsion stability. In contrast, the incorporation of non-ionic WPU enhances the flexibility and wettability of asphalt, primarily due to the contribution of its polyether structure. The characteristic peaks in unmodified cationic emulsified asphalt appear generally weaker, indicating that WPU modification significantly improves the chemical structure and overall performance of emulsified asphalt.

**Figure 3:**
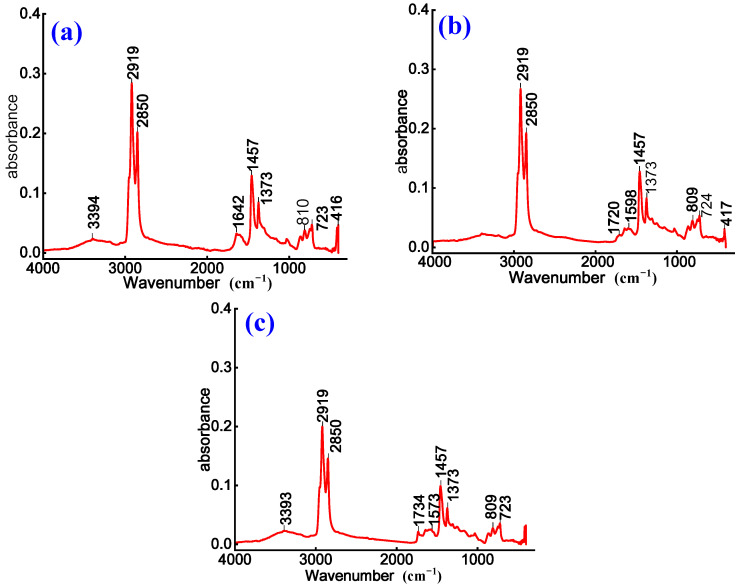
Infrared spectra of (**a**) anionic, (**b**) non-ionic WPU-modified anionic, and (**c**) anionic WPU-modified anionic emulsified asphalt.

To investigate chemical structure evolution and modification effects of anionic emulsified asphalt, non-ionic WPU-modified anionic emulsified asphalt, and anionic WPU-modified anionic emulsified asphalt, FTIR is employed to characterize three prepared samples. The corresponding results are presented in Figure 3.

As shown in Figure 3a, the absorption peak at 3394 cm^−1^ is assigned to the O–H stretching vibration, indicating the presence of polar functional groups in both emulsified asphalt and the WPU system. The absorption peaks at 2919 cm^−1^ and 2850 cm^−1^ are attributed to the antisymmetric and symmetric stretching vibrations of methylene groups, confirming the presence of alkane structures. A comparison of the spectra for WPU-modified anionic emulsified asphalt in Figure 3b,c with that of anionic emulsified asphalt in Figure 3a shows a decrease in the intensity of absorption peaks at 2919 cm^−1^, 2850 cm^−1^, 1457 cm^−1^, and 1373 cm^−1^. This reduction suggests a relative decrease in alkyl group content after modification. This is due to the dilution of saturated aliphatic components in asphalt by the introduced WPU or partial chemical reactions [14].

Furthermore, the absorption peak at 1642 cm^−1^ in Figure 3a is attributed to the bending vibration of water molecules (H-O-H) and the stretching vibration of C=C bonds, indicating the coexistence of moisture and aromatic ring structures. In Figure 3c, a weak absorption peak appears at 1734 cm^−1^, and at 1720 cm^−1^ in Figure 3b, and both correspond to the stretching vibration of carbonyl (C=O) groups. These peaks suggest that the incorporation of WPU introduces additional oxygen-containing functional groups [27], such as ester or amide groups. This effect is more prominent in Figure 3b,c, further indicating that chemical interactions between WPU and asphalt lead to the formation of ester or amide derivatives. These findings further confirm the structural stability of the asphalt matrix.

In summary, WPU incorporation not only reduces the alkyl content of emulsified asphalt but also introduces new polar functional groups, thereby enhancing the overall polarity. Non-ionic WPU improves the wettability and flexibility of asphalt, whereas anionic WPU influences emulsion dispersion and stability through the electrostatic effects of its polar groups.

### 3.3. Influence of WPU on Micromorphology and Microstructure of Emulsified Asphalt

AFM is utilized to characterize the surface morphology, microstructure, and surface roughness parameters of anionic and cationic emulsified asphalt, non-ionic WPU-modified emulsified asphalt, and anionic/cationic WPU-modified anionic/cationic emulsified asphalt. The effects of various modification formulations are systematically investigated.

#### 3.3.1. Influence of WPU on Surface Morphology of Emulsified Asphalt

**Figure 4:**
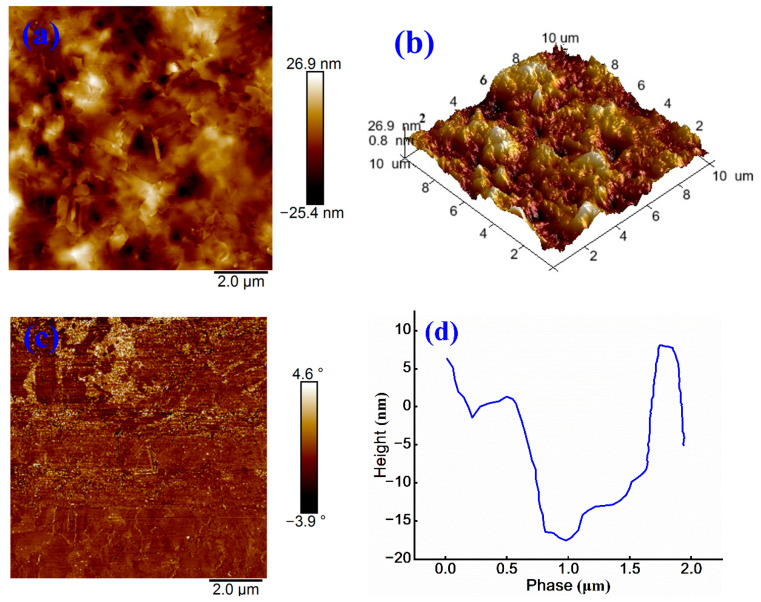
(**a**) Height image, (**b**) 3D height image, (**c**) Phase image, and (**d**) Treatment image of cationic emulsified asphalt.

The impact of cationic emulsifiers on microstructural characteristics of base asphalt is examined through AFM. The corresponding results are presented in Figure 4.

In AFM images, lighter regions indicate higher surface elevations, whereas darker regions correspond to lower elevations. This topographical pattern is also observed in the 3D morphology. As shown in Figure 4a, the sample surface exhibits a honeycomb-like structure with a relatively uniform distribution of continuous phase and a vertical height variation of approximately 52.3 nm. Figure 4b further reveals that the surface of cationic emulsified asphalt is characterized by noticeable roughness and an undulating morphology.

The phase image in Figure 4c shows a phase angle variation ranging from −3.9° to 4.6°, with a relatively uniform color distribution, indicating minimal phase separation and homogeneous surface properties. Figure 4d quantifies the surface height variation within the range of 0.5–1.5 μm, where a downward trend suggests the presence of local depressions or valleys. Beyond 1.5 μm, the surface height increases sharply, implying the existence of protrusions or peak-like structures, with a total height difference nearing 25 nm.

**Figure 5:**
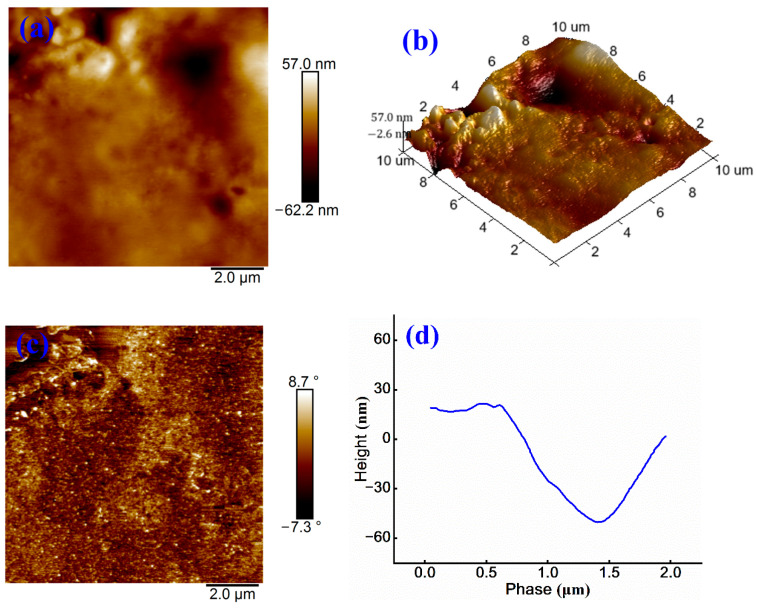
(**a**) Height image, (**b**) 3D height image, (**c**) phase image, and (**d**) treatment image of non-ionic WPU-modified cationic emulsified asphalt.

To assess the influence of non-ionic WPU on microscopic morphology of cationic emulsified asphalt, AFM characterizations are conducted on non-ionic WPU–modified samples, and the corresponding results are shown in Figure 5.

As shown in Figure 5a, the sample surface exhibits a maximum height of 57.0 nm and a minimum of −62.2 nm, resulting in a total height difference of 119.2 nm, which is increased by 66.9 nm when compared to that in Figure 4a. Uneven color distribution is observed, and brighter regions likely correspond to WPU phase, indicating that the introduction of WPU induces microphase separation among cationic groups [28]. This separation leads to the aggregation of WPU and the formation of larger phase domains, thereby increasing surface roughness. Figure 5b further reveals more distinct surface protrusions and depressions, along with reduced uniformity, supporting the occurrence of microphase separation.

In Figure 5c, the phase angle ranges from −7.3° to 8.7°, exceeding the range in Figure 4c, and is accompanied by an enhanced color contrast. This indicates greater surface hardness and more pronounced phase separation. These observations indicate greater surface stiffness and more evident phase separation, likely due to limited interaction between WPU and the cationic emulsified asphalt matrix, which reduces overall compatibility [29]. Figure 5d shows a surface height difference exceeding 60 nm, further confirming that WPU incorporation enhances surface undulations and roughness. These morphological changes influence key material properties, such as adhesion performance and mechanical stability.

**Figure 6:**
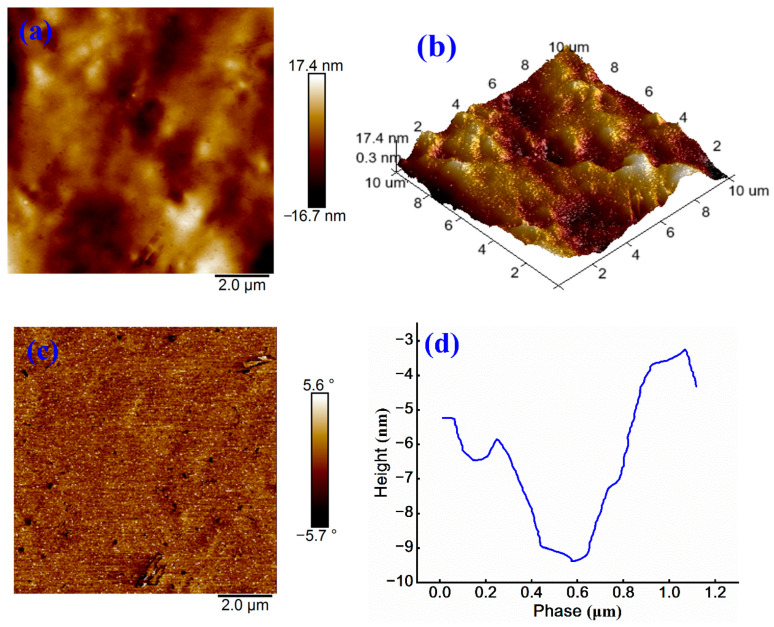
(**a**) Height image, (**b**) 3D height image, (**c**) phase image, and (**d**) treatment image of cationic WPU-modified cationic emulsified asphalt.

To further examine the influence of cationic WPU on the microstructure of cationic emulsified asphalt, a systematic analysis is conducted on the evolution of ionic structure within asphalt microstructure. AFM characterization results of modified samples are presented in Figure 6.

Figure 6a shows that the sample exhibits a bright and uniform color distribution, with a phase structure more homogeneous than those shown in Figure 4a and Figure 5a. The maximum height difference is 34.1 nm, representing the smallest surface fluctuation among the three sample groups and indicating relatively low surface roughness. Figure 6b further reveals a more uniform surface morphology, confirming that the incorporation of cationic WPU improves the microstructure of emulsified asphalt.

The modification effect of cationic WPU primarily results from the introduction of cationic groups, which regulates the molecular structure of WPU. Hydrogen bond donors (-NH, -OH) and acceptors (C=O) in the cationic WPU interact with polar groups (carboxyl and ester) in the cationic emulsified asphalt through hydrogen bonding [30], which is consistent with FTIR analysis results. These interactions enhance intermolecular forces and improve interfacial compatibility among different components. As a result, the asphalt matrix shows an improved uniformity, reduced microscopic phase separation, and decreased surface roughness.

Figure 6c,d further validate these findings. The phase angle variation remains minimal, with a balanced color distribution and smooth transitions, indicating a highly uniform surface. A phase height difference of approximately 5 nm is observed, providing further evidence of an enhanced surface uniformity and a refined microstructure. These results clearly demonstrate that cationic WPU modification significantly improves interfacial compatibility, uniformity, and surface characteristics of cationic emulsified asphalt, offering experimental support for further optimization of modification system.

**Figure 7:**
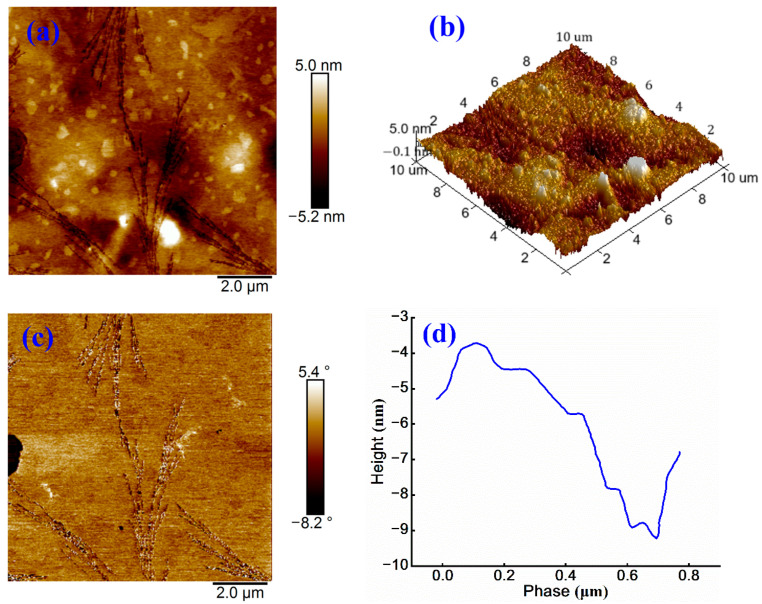
(**a**) Height image, (**b**) 3D height image, (**c**) phase image, and (**d**) treatment image of anionic emulsified asphalt.

To investigate the influence of microstructure on anionic emulsified asphalt, AFM characterization is performed on modified samples as shown in Figure 7.

Figure 7a shows that the sample displays a relatively uniform honeycomb-like morphology, characterized by a balanced color distribution and minimal bright spots. The surface height variation ranges from −5.2 nm to 5.0 nm, which is significantly lower than that observed in cationic emulsified asphalt. Figure 7b also reveals a smoother surface compared to Figure 4a. This difference is primarily attributed to the charge properties and particle sizes of anionic and cationic emulsifiers [31]. In anionic emulsified asphalt, negatively charged particles repel each other, promoting a more uniform dispersion and resulting in a smoother surface. In contrast, positively charged particles in cationic emulsified asphalt interact electrostatically with negatively charged mineral aggregates, causing particle aggregation and greater surface undulations. Moreover, as shown in Figure 5, the particles in anionic emulsified asphalt are generally smaller and more uniformly dispersed within the matrix, thereby reducing the overall surface roughness.

Figure 7c presents a phase angle variation ranging from −8.2° to 5.4°, along with a relatively a smooth color transition, indicating minimal variations in surface physical properties. A low degree of phase separation is detected. The height variation in Figure 7d approaches 6 nm, reflecting reduced surface roughness, which is consistent with the previous analytical results.

**Figure 8:**
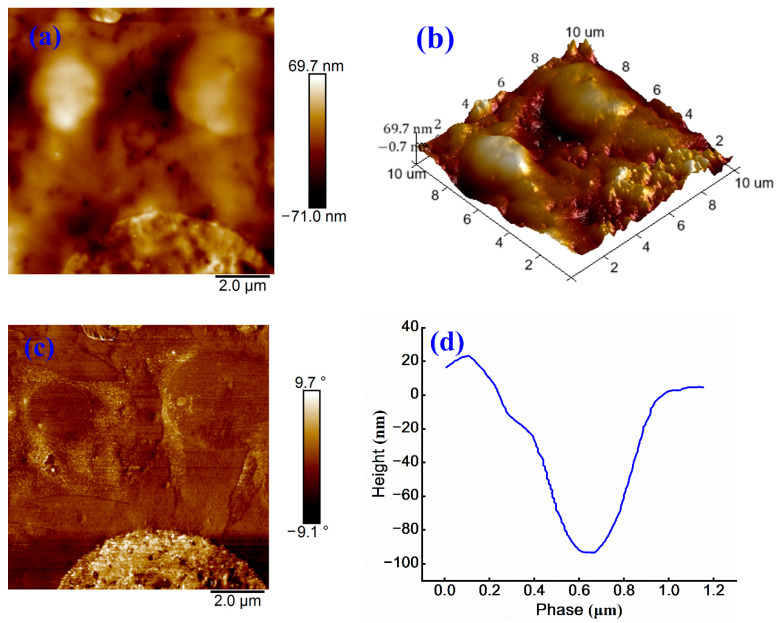
(**a**) Height image, (**b**) 3D height image, (**c**) phase image, and (**d**) treatment image of non-ionic WPU-modified anionic emulsified asphalt.

To investigate modification effect of non-ionic WPU on the microstructure of anionic emulsified asphalt, AFM characterization is conducted on the modified samples. The corresponding results are shown in Figure 8.

Figure 8a shows a reduced honeycomb structure, accompanied by an increase in both the number and area of bright regions. The maximum surface height point reaches 69.7 nm, while the minimum is −71.0 nm, respectively, yielding a total height variation of 140.7 nm. Compared to Figure 5a, the surface roughness increases by 21.5 nm, indicating that the roughness of WPU-modified anionic emulsified asphalt exceeds that of cationic emulsified asphalt. This increase is likely caused by weak interactions between WPU and anionic groups, leading to WPU aggregation, non-uniform dispersion, and elevated surface roughness [32]. This observation is further supported by Figure 8b.

Figure 8c displays a broader range of phase angle variations when compared to Figure 7c, indicating an enhanced phase separation after the addition of WPU into anionic emulsified asphalt. The height difference in Figure 8d is approximately 110 nm, and the surface appears uneven, further confirming poor compatibility of WPU within anionic emulsified asphalt system.

**Figure 9:**
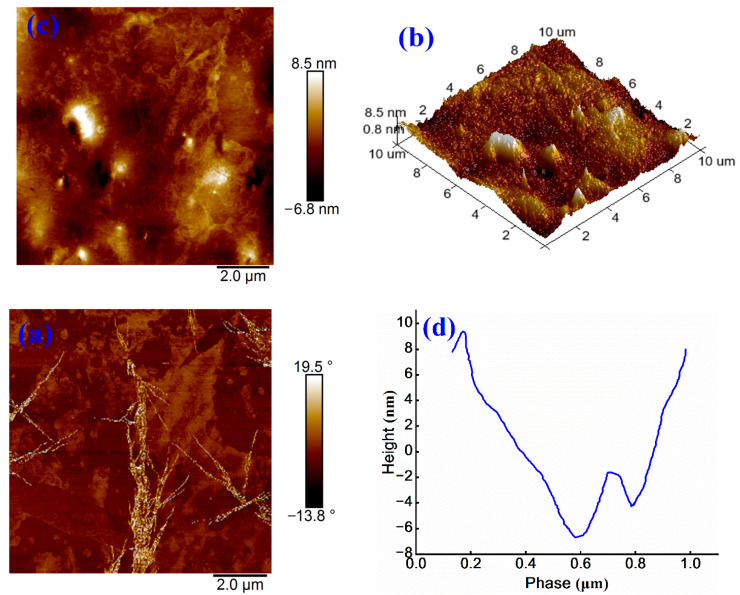
(**a**) Height image, (**b**) 3D height image, (**c**) phase image, and (**d**) treatment image of anionic WPU-modified anionic emulsified asphalt.

To examine the effect of anionic WPU modification on the microstructure of anionic emulsified asphalt and to facilitate the comparison with previously modified samples, AFM tests are employed for surface characterization. The detailed results are presented in Figure 9.

As shown in Figure 9a, the continuous and dispersed phases of the sample are uniformly distributed, and only a few bright regions are observed. The maximum surface height variation is measured at 15.3 nm. Compared to Figure 8a, an increased number of honeycomb-like structures increases and exhibits a more balanced distribution. Further analysis of Figure 9b reveals a more uniform surface morphology when compared to 3D images of other anionic emulsified asphalt samples, indicating that the incorporation of anionic WPU enhances microstructural homogeneity.

Figure 9c presents a phase angle range from −13.8° to 19.5°, with minimal overall color variation. However, a small “dendritic” bright region is observed, indicating a localized area of significant variation in physical properties within the scanned region. This phenomenon may be attributed to sample selection or the preparation process [33]. As shown in Figure 9d, the phase height difference is approximately 16 nm, which is considerably lower than that in Figure 8d and comparable to the value in Figure 7d. These findings suggest that anionic WPU achieves a more effective modification of anionic emulsified asphalt than non-ionic WPU. Overall, these findings strongly support the effectiveness of anionic WPU in enhancing interfacial compatibility, surface uniformity, and microstructural performance of anionic emulsified asphalt.

#### 3.3.2. Influence of WPU on Surface Roughness of Emulsified Asphalt

Although the honeycomb structure partially captures surface undulations, its characterization range is limited, making it difficult to comprehensively describe the overall morphology and surface microfeatures. In contrast, root mean square (RMS) roughness, a key parameter for quantifying surface irregularities [34,35], is calculated using Equations (5) and (6). The RMS roughness of both base emulsified asphalt and WPU-modified emulsified asphalt is quantified, providing a more precise representation of microstructural characteristics and local heterogeneity. Therefore, introducing RMS roughness as a primary characterization metric enhances the understanding of surface morphology and its influence on the functional performance of asphalt materials.

(5) $$R_{q}={\left[\frac{\iint {\left[h\left(x,y\right)-h_{0}\right]}^{2}dA}{\iint dA}\right]}^{1/2}$$

(6) $$h_{0}=\frac{\iint h\left(x,y\right)dA}{\iint dA}$$

where *R_q_*—RMS roughness, nm; h(x,y)—height function, nm; *A*—scanned area; $h_{0}$—reference height, nm.

The RMS roughness of sample is calculated using NanoScope Analysis 3.0 software, and the results are presented in Table 7.

As shown in Table 7, the lowest surface roughness of 1.75 nm is observed in anionic emulsified asphalt, whereas the highest value (19.7 nm) is recorded for non-ionic WPU-modified anionic emulsified asphalt. The incorporation of WPU generally increases the surface roughness of samples. However, RMS roughness of cationic emulsified asphalt modified with cationic WPU decreases to 4.73 nm. This behavior is attributed to the intrinsic leveling and filling properties of polyurethane, which allow it to fill microscale surface irregularities and produce smoother surfaces. Additionally, WPU covers or fills uneven substrate areas, thereby reducing surface granularity and overall roughness.

Upon addition to emulsified asphalt, WPU cures and interacts with phenolic and nitrogen-, oxygen-, and hydrogen-containing functional groups in asphalt through hydrogen bonding and other intermolecular interactions [36,37]. These interactions enhance the compatibility between WPU and asphalt, promoting uniform dispersion of WPU within the asphalt matrix. Consequently, the modifying effects of WPU are enhanced, improving the flexibility, viscosity, and adhesiveness of the asphalt mixture. As these properties improve, surface roughness decreases due to the formation of a more uniform and continuous asphalt coating, which reduces surface irregularities.

AFM test results show that anionic WPU-modified anionic emulsified asphalt exhibits the lowest surface roughness, indirectly indicating superior compatibility between anionic WPU and base asphalt. However, the impact of WPU addition on surface roughness remains uncertain. To optimize the modification effect and achieve controlled surface roughness, further comprehensive studies are required, including selection of suitable WPU types, dosage optimization, and refinement of emulsified asphalt formulation and preparation conditions.

## 4. Conclusions

Based on the above experimental results and discussion, the main conclusions are summarized as follows:

(1) This study compares three ionic types of WPU based on fundamental performance indicators, clarifying the influence of ionic structure differences on the properties of emulsified asphalt. Compared to base emulsified asphalt, all WPU-modified samples exhibit a reduced penetration, an increased softening point, and a decreased ductility. Among them, the non-ionic WPU-modified system enhances material hardness and high-temperature stability but results in the most pronounced low-temperature brittleness, while the cationic WPU maintains a balance between high-temperature stability and low-temperature flexibility.

(2) The intrinsic effect of WPU’s ionic structure on the storage stability of emulsified asphalt is identified. WPU modification reduces storage stability, which is primarily affected by the pH value and particle size of WPU. The cationic WPU-modified emulsified asphalt shows the poorest stability with a residue of 0.7% on the sieve, whereas the anionic WPU-modified sample exhibits the best stability with a residue of 0.4% on the sieve.

(3) The modification mechanisms by different ionic WPU structures are systematically investigated through FTIR analysis. The results indicate that ionic WPUs alter the functional group composition and polarity distribution of emulsified asphalt via a combination of physical blending and partial chemical reactions. Cationic WPU promotes the formation of ester groups and amide bonds to improve structural stability, non-ionic WPU enhances the flexibility and wettability, and anionic WPU improves the dispersion.

(4) The microstructural and interfacial morphological effects of ionic WPU on emulsified asphalt are examined using AFM. The results confirm that WPU significantly influences the microstructure and surface roughness of emulsified asphalt. Anionic WPU demonstrates the best dispersion, forming fine and uniformly distributed particles with the lowest surface roughness (5.655 nm, Ra = 1.63 nm). Cationic WPU induces chain-like structures with good curing performance, while non-ionic WPU increases the toughness but leads to greater surface undulation and reduces the uniformity. Distinct regulatory effects on interfacial morphology are observed across different WPU ionic types.

## 5. Future Directions

This study investigates the properties and compatibility of different ionic WPU-modified emulsified asphalt from both macro- and microscale perspectives, elucidating the modification mechanisms and providing a theoretical foundation for WPU applications in emulsified asphalt. However, current research still exhibits limitations, including that key macroscopic performance indicators (e.g., rheological and aging properties) need to be sufficiently explored, and a comprehensive analysis of WPU’s influence on asphalt mixture performance is lacking. Future research should focus on the following directions:

(1) Expanding the research scope. Shifting from material properties to performance evaluation of asphalt mixtures under actual engineering conditions to enhance its applicability.

(2) Enhancing durability assessment. Incorporating critical durability tests (e.g., moisture damage, aging, and freeze–thaw resistance) to evaluate long-term performance.

(3) Deepening mechanistic understanding. Integrating multiscale characterizations and computational models to clarify the interfacial interaction mechanisms between WPU and emulsified asphalt, guiding material design optimization.

These advancements are expected to improve the overall performance of WPU-modified emulsified asphalt and facilitate its broader engineering applications.

## Figures and Tables

**Table 1:** Basic technical indicators of 70# base asphalt.

Technical Indexes	Unit	Test Results	Standard Requirements	Experimental Methods
Penetration (25 °C, 5 s, 100 g)	0.1 mm	70	60–80	T0604
Ductility (10 °C)	cm	18	≥15	T0605
Softening point	°C	50	≥47	T0606

**Table 2:** Parameters of slow-cracking and fast-setting anionic and cationic emulsifiers.

Parameter	Specification Requirements		Test Results
Appearance	Cationic	Brown viscous liquid	
	Anionic	Light yellow liquid	
Active substance content	Cationic	70 ± 2	69
	Anionic		70
pH value	Cationic	6–8	7.6
	Anionic		
Free amine content (%)	Cationic	≤2	1.25
	Anionic		1.23
Density (g/cm^3^)	Cationic	1.0	1.0
	Anionic	1.05	1.05
Effective ingredient (%)	Cationic	50	50
	Anionic	40	40

**Table 3:** Technical index of hydrochloric acid and sodium hydroxide.

Test Specimens	Acid	Alkali
Chemical formula	HCl	NaOH
Molecular weight	36.5	40.0
Concentration/%	36–38	5%
Color	Colorless	White
Density/(g/cm^3^)	1.18	1.05

**Table 4:** Performance index of cationic/anionic emulsified asphalt.

Test Items	Quality Indicators		Measured
Residual on sieve (1.18 mm)/%	Cationic	<0.1	0.005
	Anionic		0.003
Particle Polarity	Cationic	Cationic	Cationic
	Anionic	Anionic	Anionic
Particle size/μm	Cationic	≤7	4.41
	Anionic		4.30
Standard viscosity/(Pa.s)	Cationic	8–25	12
	Anionic		15
Storage stability (1d, 25 °C)/%	Cationic	<1	0.4
	Anionic		0.3
Content/%	Cationic	≥60	63.7
	Anionic		62.8
Penetration/(0.1 mm)	Cationic	40–120	75
	Anionic	50–300	79
Softening point/°C	Cationic	≥42	52.9
	Anionic		53.1
Ductility/cm	Cationic	≥40	52.5
	Anionic		90.3

**Table 5:** Performance indexes of different ionic WPUs.

Type	Designation	Solids Content (%)	pH Value	Viscosity Value (mPa.s)	Color	Specific Gravity (g/cm^3^)
A	Cationic WPU	38 ± 1	3–7	<200	Milky white	1.06 ± 0.02
B	Anionic WPU	30 ± 1	6–9	≥100		1.05
				20 °C		
C	Non-ionic WPU		6–8	>100		

**Table 6:** Performance indexes of WPU-modified emulsified asphalt samples.

Test Items	Cationic Emulsified Asphalt	Cationic WPU + Cationic Emulsified Asphalt	Non-ionic WPU + Cationic Emulsified Asphalt	Anionic Emulsified Asphalt	Anionic WPU + Anionic Emulsified Asphalt	Non-ionic WPU + Cationic Emulsified Asphalt
Residual on sieve (1.18 mm)/%	0.005	0.006	0.003	0.003	0.015	0.002
Particle polarity	C	C	C	A	A	A
Standard viscosity /(Pa.s)	12	16	15	15	17	16
Storage stability (1d, 25 °C)/%	0.4	0.7	0.5	0.3	0.4	0.5
Solid content (%)	65	63.7	62.8	65	62.8	62.8
Penetration/0.1 mm	75	60.3	59.3	79	70	63.3
Softening point/°C	52.9	54.1	53.8	53.1	54	53.9
Ductility (15 °C)/cm	52.5	43.2	36.4	90.3	44.7	28.7

**Table 7:** Rq values of different emulsified asphalt samples.

Sample Name	Cationic Emulsified Asphalt	Cationic WPU + Cationic Emulsified Asphalt	Non-ionic WPU + Cationic Emulsified Asphalt	Anionic Emulsified Asphalt	Anionic WPU + Anionic Emulsified Asphalt	Non-ionic WPU + Cationic Emulsified Asphalt
Surface Rq/nm	7.32	4.73	13.6	1.75	1.63	19.7

## Data Availability

The original contributions presented in the study are included in the article, further inquiries can be directed to the corresponding authors.
